# Microsatellite DNA Analysis for Diversity Study, Individual Identification and Parentage Control in Pig Breeds in Poland

**DOI:** 10.3390/genes12040595

**Published:** 2021-04-19

**Authors:** Anna Radko, Grzegorz Smołucha, Anna Koseniuk

**Affiliations:** Department of Animal Molecular Biology, National Research Institute of Animal Production, Krakowska Street 1, 32-083 Balice, Poland; grzegorz.smolucha@iz.edu.pl (G.S.); anna.koseniuk@iz.edu.pl (A.K.)

**Keywords:** pig, biodiversity, STR, individual identification, parentage

## Abstract

Swine DNA profiling is of high importance for animal identification and parentage verification. The aim of this study was to test a set of 14 microsatellite (STR) markers recommended by ISAG for parentage verification in Polish Landrace (PL, *n* = 900), Polish Large White (PLW, *n* = 482), Pulawska (PUL, *n* = 127), and Duroc pigs (DU *n* = 108). The studied breeds showed a medium level of genetic differentiation. The average value of heterozygosity and degree of polymorphism (PIC) were above 0.5 for the studied breeds, except for the DU breed (PIC = 0.477). The population inbreeding coefficient indicates an absence of inbreeding in the studied breeds (an average value of F_IS_ = 0.007). The cumulative power of discrimination for all breeds reached high values close to 1.0, while the probability of identity (P_ID_) was low, with P_ID_ values ranging between 10^−9^ (for DU) and 10^−12^ (for PLW). The cumulative exclusion probability for PE_1_ and PE_2_ showed that the parentage can be confirmed with a probability of from 92.75% to 99.01% and from 99.49% to 99.97%, respectively.

## 1. Introduction

Pork is the most frequently chosen meat by Polish consumers. Poland, along with Austria and Spain, is leading in pork consumption, while in terms of the pork production, Poland ranks 4th, after Germany, Spain and France [1]. High quality standards of pork production are maintained by the implementation of breeding work. In Poland, the Polish Pig Breeders and Producers Association “POLSUS” controls the population of breeding pigs and the implementation of the breeding program. Currently, the National Breeding Program includes the following pig breeds: Polish Large White (PLW), Polish Landrace (PL), Pulawska (PUL), Duroc (DU), Hampshire, and Pietrain. These breeds are used in crossbreeding for the commercial production of fattening pigs in Poland [2]. A successful breeding relies on breeding progress, which can be achieved only if individual identification as well as parentage data are consistent with breeding documentation. In Poland, the systematic control of the parentage of pigs has been carried out since 1977, initially on the basis of blood groups, and since 2016, based on DNA. In the 1990s, the International Society for Animal Genetics (ISAG) officially released recommendations to conduct the parentage verification of farm animals using microsatellite markers or short tandem repeats (STR). The STR still constitute the huge group of markers used in the study of genetic structure and variability, as well as in that of parentage control for different species of farm animals [3,4,5,6,7], including pigs [8,9,10,11,12,13,14]. At the ISAG conference in 2012, a microsatellite panel consisting of the following 24 markers was proposed for the first time: IGF1, S0002, S0005, S0026, S0068, S0090, S0101, S0155, S0178, S0215, S0225, S0226, S0227, S0228, S0355, S0386, SW024, SW072, SW240, SW632, SW857, SW911, SW936, and SW951 [15]. In 2014, a recommended list of markers was modified and divided into core and additional panels. The core panel consisted of 15 microsatellite loci: S0005, S0090, S0101, S0155, S0227, S0228, S0355, S0386, SW24, SW240, SW72, SW857, SW911, SW936, and SW951; the additional panel included 7 microsatellites: IGF1, S0002, S0026, S0215, S0225, S0226, and SW632 [16].

In the study, we analyzed the polymorphism of DNA microsatellite markers of the core STR panel in the Polish pig breeds PLW, PL, and PUL, as well as in one foreign breed–DU. The PLW and PL breeds were created as a result of many years of breeding work by crossing the following breeds: the Large and Medium White English with the German Noble, and the Deutsches veredeltes Landschwein (Improved German Landrace) with the Svensk Lantras (Swedish Landrace). These breeds are currently of the greatest economic importance and constitute a maternal component in the commercial crossing of pigs [17]. The native PUL breed was created as a result of crossing local pigs with the Berkshire breed. The Pulawska breed, known as the “pigeon pig” before World War II, is used for commercial crossing [18]. On the other hand, one of the most frequently bred paternal breeds in Poland is the American Duroc breed, which produces high-quality meat and is an important component in breeding programs [19].

**Figure 1:**
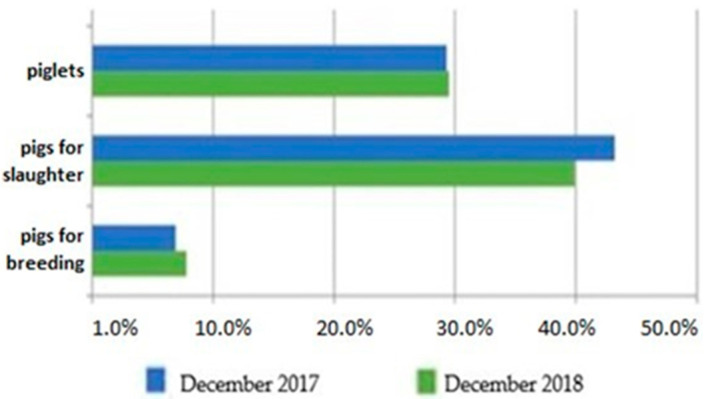
Pig population structure in December 2017 and 2018. http:/stat.gov.pl (accessed on 2 October 2019).

The Pork Quality System (PQS)—developed by the Polish Pig Breeders and Producers Association (POLSUS) and the Polish Meat Association—assumes planned crossbreeding as a highly effective method in pig production for the improvement of the carcass and for high-quality pork. The genetic potential of the breeds PLW, PL, PU and DU was utilized as the maternal (PLW, PL, and PU) and paternal (DU) components due to their high-carcass meat content, low fatness, adequate meat quality, and favorable intramuscular fat (IMF) levels [2]. At the beginning of June 2020, the swine population in Poland was over 11.4 million head, about 40% of which were fattening pigs [2]. The structure of the pig population is shown in Figure 1 [1].

The aims of this study were to present the genetic structure of selected major breeds of pigs in Poland and to determine the usefulness of a panel of 14 STR markers for individual identification and parentage control studies.

## 2. Materials and Methods

### 2.1. Material

Blood samples were collected from pigs subjected to the routine parentage testing at the National Research Institute of Animal Production from 2017 to2020. A total of 1617 pigs were investigated, representing four breeds: Polish Landrace (PL, *n* = 900), Polish Large White (PLW, *n* = 482), Pulawska (PUL, *n* = 127), and Duroc (DU, *n* = 108).

DNA was extracted from blood samples using the Sherlock AX Kit (A&A Biotechnology, Gdynia, Poland), following the manufacturer’s protocol. The extracts were quantified with a NanoDrop 2000 spectrophotometer (Thermo Scientific, Wilmington, DE, USA).

The analysis made use of 14 STR—recommended by ISAG as the core panel for the identification of individuals and parentage testing in pigs. The markers and used primer sequences are described in Table 1.

### 2.2. Methods

The 14 STR loci were amplified in one multiplex panel using Type-it Microsatellite PCR Kit (Qiagen Inc., Hilden, Germany) reagents and fluorescently labeled primers (Table 1). The PCR reaction was performed on the Veriti^®^ Thermal Cycler amplifier (Applied Biosystems, Foster City, CA, USA) using the following thermal profile: 5 min of initial DNA denaturation at 95 °C, followed by 28 cycles of denaturation at 95 °C for 30 s, annealing at 57 °C for 90 s, elongation of starters at 72 °C for 30 s, and a final elongation of starters at 60 °C for 30 min. The analysis of the obtained PCR products was performed using an ABI 3130xl capillary sequencer (Applied Biosystems, Foster City, CA, USA). The amplified DNA fragments were subjected to electrophoresis in 7% denaturing POP-7 polyacrylamide gel in the presence of a standard length of 500 LIZ (ThermoFisher Scientific) and a reference sample. The results of the electrophoretic separation were analyzed automatically using the GeneMapper^®^ Software 4.0 (Applied Biosystems, Foster City, CA, USA).

### 2.3. Data analysis

The observed heterozygosity (H_O_), expected heterozygosity (H_E_), and inbreeding coefficient (F_IS_) for each marker were estimated in each breed and calculated according to Nei and Roychoudhury [20], and Wright [21]. The Hardy–Weinberg equilibrium (HWE) of the 14 STR loci was tested with an exact test, using an algorithm based on Markov Chain Monte Carlo methods [22]. A Bonferroni correction was performed using the R Statistical Package [23].

The genetics parameters were calculated:

- Polymorphic information content—PIC [24];
- Power of discrimination—PD [25];
- Probability of identity—P_ID_ [26];
- Probability of parentage exclusion for each locus—when the genotypes of one and both parents are known (PE_1_ and PE_2_)—and the cumulative probability of parentage exclusion (CPE), according to Jamieson’s (1994) formula [27].

The statistical analysis was carried out by IMGSTAT software, ver. 2.10.1 (2009), which supports the laboratory of the National Research Institute of Animal Production.

## 3. Results

**Figure 2:**
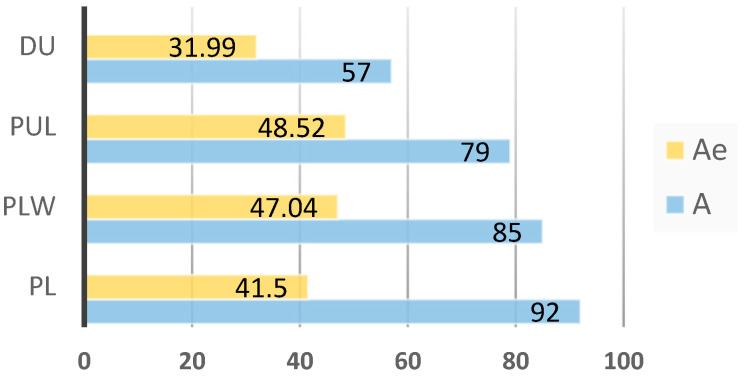
Number of alleles (A) and effective number of alleles (Ae) for each breed.

For the 14 STR loci analyzed, 101 alleles were detected in the 4 breeds studied (Table 2). The highest number of alleles was identified in SW857 and SW936 (10 alleles in both loci), and the smallest number (3 alleles) in the S0227 locus. Alleles established for the studied breeds in particular loci are presented in Table 2. The PL breed had the highest number of alleles, for which 92 alleles were determined, while the highest effective number of alleles occurred in the PUL breed (48.52 alleles). The lowest number of alleles and the lowest effective number of alleles (57 and 31.99, respectively) were established for the DU breed (Figure 2). The number of alleles and effective number of alleles averaged across loci and breeds were 5.62 and 2.90, respectively.

### 3.1. Diversity Analysis

The genetic variability of the studied breeds in 14 loci is presented in Table 3. From among the markers, the S0090, S0155, SW24, SW72, SW857, and SW936 loci were found to have high Ho values (above 0.5) for all the breeds studied. For the Polish breeds PL, PLW, and PUL, the highest value of Ho > 0.8 was observed in locus SW857, while for the DU breed, the highest value of Ho > 0.7 was found in the SW24 and SW72 loci. The lowest Ho < 0.4 values for all 4 breeds were found in locus S0227, while the lowest heterozygosity (0.213) was observed in S0355 in the DU breed. The means across loci for the expected and observed heterozygosities were 0.599 and 0.605, respectively (Table 3). Estimates of within-breed genetic diversity are summarized in Table 4. The highest average heterozygosity was found for PLW (Ho = 0.632) and the lowest for DU (Ho = 0.546). The values of Ho and He were similar in most loci, while the mean value of inbreeding coefficient was 0.007, ranging from -0.051 (DU) to 0.054 (PLW). In four cases, the inbreeding coefficient reached quite high positive F_IS_ values for the PLW breed in the S0155 locus (F_IS_ = 0.108), and for the DU in S0355, S0385, and SW24, (F_IS_ = 0.276, 0.274 and 0.105, respectively). In these cases, deviation from the Hardy–Weinberg equilibrium was also observed (Table 3). In total, there were 18 deviations from the HWE at the *p*-value of less than 0.01 and 0.001 in the target pig population. Only in loci S0227, S0228, SW857, and SW911 was there no deviation from the HWE equilibrium noted. The *p*-value of HWE for the PL breed in SW936, and for the DU in SW386, SW936, and SW951 was lower than 0.05; however, when a Bonferroni correction was applied, the *p*-value increased to above 0.05.

### 3.2. Parentage Testing and Individual Identification

The parameters for determining the suitability of the analyzed STR panel for the identification and parentage testing of each breed are presented in Table 3. For the PL, PLW, and PUL breeds, polymorphism exceeding 0.5 was observed in the majority of loci, except in the S0227, S0355, SW911, and SW951 loci. The highest values (PIC > 0.7 for PLW, and PIC > 0.8 for PL and PUL) were observed in the SW857 locus. The lowest polymorphism was found in the Duroc, for which there were as many as 6 loci with a value of PIC < 0.5, and 4 loci with the value PIC < 0.3 (Table 3).

The mean PIC values for the studied breeds varied between 0.447 (DU) and 0.623 (PLW) (Table 4). The mean PD value for 14 STR, calculated for all pig breeds together, was 0.771 (Table 3). The power of discrimination for the whole set of STR, and for each of the breeds, shows the high values of 0.999999982923212 (DU) and 0.999999999998066 (PLW).

On the basis of P_ID_ calculated for each locus (Table 3), we estimated the cumulative probability of identity for the 14 STR loci together and obtained values as low as 2.0 × 10^−12^ and 6.9 × 10^−09^ for PLW and DU, respectively (Table 4). The panel of 14 microsatellite markers was assessed for their power of exclusion to test parentage in the four breeds of pigs. The probabilities of exclusion were calculated for two hypothetical situations—with one parental genotype available (PE_1_) and two parental genotypes available (PE_2_). The probability of exclusion for one parent available (PE_1_) ranged between 0.02 (S0355 in DU) and 0.539 (SW857 in PL), and when two parents were available (PE_2_), between 0.105 (S0355 in DU) and 0.703 (SW857 in PL), across different markers and breeds. The cumulative exclusion probability for PE_1_ and PE_2_ varied from 0.9275 (DU) to 0.9909 (PLW), and from 0.9949 (DU) to 0.99978 (PLW), respectively (Table 4).

## 4. Discussion

The individual identification of pigs, most often performed in parentage control and an important element of breeding work, is aimed at achieving high-quality pork [28,29]. The identification of pigs is also important in keeping food safe for animals and consumers, in the case of adulteration and frauds [28,29,30,31]. To carry out routine individual identification tests, it is necessary to know the genetic structure of the pig population, especially those that play an important role in pig breeding and production. The conducted studies determined polymorphism in 14 STR loci recommended for pig identification in four major breeds of pigs in Poland. A total of 101 alleles were identified for the breeds and loci, while the total mean and the effective number of alleles at the loci were at 7.21 and 2.90, respectively. The high effective numbers of 48.5 and 47.0 alleles were found in the PUL and PLW breeds, respectively. The lowest effective number of alleles was observed in the DU breed (31.99). The effective number of alleles translated into the degree of heterozygosity in those breeds, which was the highest in the PLW and PUL (Ho = 0.632 and Ho = 0.619, respectively), and the lowest in the DU breed (Ho = 0.546). The lowest effective number of alleles in the locus and the degree of heterozygosity were also observed in the DU breed in Portugal [10]. Higher values for this breed were found in Thailand (Ho = 0.627) [29]. The heterozygosity coefficient calculated on the same 12 studied in the STR markers in five breeds of pigs in the Ukraine was at a similar level. The Ho values were from 0.42 to 0.67, and in the population of pigs of five commercial breeds in Brazil, the mean Ho values ranged from 0.42 to 0.57 [11,12]. In the study population, the values of the inbreeding coefficient reached positive values F_IS_ > 1 only for two loci in the PLW and DU breeds, and positive values F_IS_ > 2 for two loci in the DU breed. The mean value of F_IS_ for PL and DU was negative, and assumed a positive low value of F_is_ = 0.007 for four breeds, which proves the lack of inbreeding in the studied pig population. For eleven European pig breeds and 17 STR loci, a slightly higher value of F_is_ = 0.052 was obtained [8].

PIC, PD, and P_ID_ are important indicators for the polymorphism of the genetic markers used in individual identification. STR markers with PIC values exceeding 0.5 were considered highly informative [24,32]. In the analyzed population, the mean degree of polymorphism, which was calculated based on 14 STR, was higher than 0.5 for the PL and PUL breeds, and higher than 0.6 for the PLW breed. Only the DU breed was found to have a lower degree of polymorphism, with a PIC value of 0.477. The polymorphic information content takes into account the number of alleles and their frequency. A high frequency of some alleles was observed in the DU breed, and as many as five loci have an effective number of alleles at the locus lower than two alleles (Table 2). Other studies conducted for different breeds of pigs, depending on the STR used, showed mean PIC values ranging from 0.4 to 0.8 [10,13,14,28,29,31]. The use of a set of markers with a high power of discrimination enables individual identification. The power of discrimination is the probability that two individuals randomly selected from a population will have a different set of traits. The higher the power of discrimination, the more polymorphic the markers are. This is confirmed by our research in which the highest PD value = 0.9999999999998066 was obtained for the PLW breed with the highest polymorphism (Ho = 0.632; PIC = 0.623), and the lowest PD value = 0.9999999982923212 for the DU breed with the lowest polymorphism (Ho = 0.546; PIC = 0.477). The probability of identity shows the probability of finding two unrelated, randomly selected individuals in the population that will have the same genotype. When we used the selected 14 STR, the probability of finding two individuals on the same profile was 10^−12^ for PL, 10^−11^ for PL and PUL, and 10^−9^ for the DU breed. For the DU breed in Taiwan, a higher P_ID_ of 10^−7^ was obtained, based on 13 STR [14]. Likewise for this breed in China, based on 12 and 17 loci P_ID_ were at levels 10^−4^ and 10^−5^, respectively. The other five Chinese breeds had P_ID_ values based on 17 loci ranging from 10^−9^ to 10^–15^ [31]. For two pig populations in Korea, the P_ID_ calculated based on 13 STR took the values 9.87 × 10^−14^ and 1.03 × 10^−9^ [28]. Similarly, the probability of identity using 11 STR for the Polish Zlotnicka White and Zlotnicka Spotted amounted to 3.12 × 10^−10^ and 5.92 × 10^−10^, respectively [33]. The probability of parentage exclusion, calculated in cases when the genotypes of one and both parents are known (PE_1_ and PE_2_), is a direct indicator that determines the usefulness of DNA markers for verifying the origin of the indicated parents. The cumulative probability of parentage exclusion for all breeds of 14 STR used in this study achieved a CPE_1_ above 0.98 and CPE_2_ above 0.999, except for DU (CPE_1_ = 0.9270, CPE_2_ = 0.995). It can, therefore, be concluded that if the genotype of one of the parents is known, we can confirm the origin of the PL, PLW, and PUL breeds with 98% probability, and the DU breed with 90% probability. On the other hand, knowing the genotype of both parents increases the probability to 99.9% for the PL, PLW and PUL breeds, and up to 99.5% for the DU breed. CPE2 of 99.9% was also demonstrated for pigs in Taiwan [14], for the Polish Zlotnicka pigs [33], and for the Austrian pigs [34].

## 5. Conclusions

The studies showed an average degree of genetic differentiation in the PL, PLW, and PUL breeds, while a limited degree of polymorphism (below 50%) was observed in the DU breed. The indicators for assessing the usefulness of the STR panel studied indicate its suitability for routine tests in the tested breeds. However, lower parameters obtained for the DU breed indicate the need to monitor ongoing changes. Our results provide baseline data for monitoring pig diversity and breed management, which is necessary for the identification of pork and pork products for consumers.

It also seems reasonable to prepare an additional panel of markers, which can be used in cases where the 14 STR recommended by ISAG would be insufficient for individual identification and parentage control.

## Figures and Tables

**Table 1:** The list of used the ISAG-recommended markers with primer information.

locus	Size Range	Dye	Primer (5′-3′): Forward Primer (5′-3′): Reverse
S0090	244–251	FAM	CAAGACTGCCTTGTAGGTGAATA GCTATCAAGTATTGTACCATTAGG
S0101	197–216	FAM	GAATGCAAAGAGTTCAGTGTAGG GTCTCCCTCACACTTACCGCAG
S0155	150–166	PET	TGTTCTCTGTTTCTCCTCTGTTTG AAAGTGGAAAGAGTCAATGGCTAT
S0227	231–256	NED	GATCCATTTATAATTTTAGCACAAAGT GCATGGTGTGATGCTATGTCAAGC
S0228	222–249	VIC	GGCATAGGCTGGCAGCAACA AGCCCACCTCATCTTATCTACACT
S0355	243–277	PET	TCTGGCTCCTACACTCCTTCTTGATG TTGGGTGGGTGCTGAAAAATAGGA
S0386	156–174	NED	GAACTCCTGGGTCTTATTTTCTA GTCAAAAATCTTTTTATCTCCAACAGTAT
SW24	96–121	PET	CTTTGGGTGGAGTGTGTGC ATCCAAATGCTGCAAGCG
SW72	100–116	VIC	ATCAGAACAGTGCGCCGT TTTGAAAATGGGGTGTTTCC
SW240	96–115	FAM	AGAAATTAGTGCCTCAAATTGG AAACCATTAAGTCCCTAGCAAA
SW857	144–160	VIC	TGAGAGGTCAGTTACAGAAGACC GATCCTCCTCCAAATCCCAT
SW911	153–177	FAM	CTCAGTTCTTTGGGACTGAACC CATCTGTGGAAAAAAAAAGCC
SW936	80–117	NED	TCTGGAGCTAGCATAAGTGCC GTGCAAGTACACATGCAGGG
SW951	125–133	FAM	TTTCACAACTCTGGCACCAG GATCGTGCCCAAATGGAC

**Table 2:** Number of alleles per *locus* (N), allele range, number of alleles per *locus* (A) and effective number of alleles per *locus* (Ae) for each breed.

Locus	N	Breeds	Size Range	A	Ae
S0090	7	PL	234–254	7	2.90
		PLW	244–254	5	4.05
		PUL	244–254	5	2.81
		DU	246–252	4	2.62
S0101	6	PL	200–218	5	2.73
		PLW	200–218	5	3.33
		PUL	200–218	6	3.40
		DU	212–218	4	1.49
S0155	7	PL	152–168	6	2.86
		PLW	152–166	5	2.56
		PUL	152–166	5	2.88
		DU	158–166	3	2.56
S0227	3	PL	232–256	3	1.52
		PLW	232–256	3	1.42
		PUL	232–256	3	1.43
		DU	232–256	2	1.54
S0228	7	PL	218–244	7	2.65
		PLW	218–244	6	5.21
		PUL	218–244	6	2.61
		DU	222–232	3	1.51
S0355	7	PL	247–275	7	2.23
		PLW	247–275	5	3.42
		PUL	247–275	5	1.82
		DU	247–275	4	1.25
		PL	157–177	8	3.58
S0386	8	PLW	157–177	7	2.94
		PUL	157–177	6	3.02
		DU	167–177	3	1.93
SW24	9	PL	98–122	7	2.86
		PLW	98–122	8	3.16
		PUL	98–118	6	4.44
		DU	98–122	7	3.94
SW72	6	PL	103–119	6	2.86
		PLW	103–119	6	4.00
		PUL	103–119	6	3.11
		DU	103–119	4	3.21
SW240	9	PL	98–116	9	2.82
		PLW	98–118	8	2.73
		PUL	98–116	8	3.00
		DU	100–116	5	2.65
SW857	10	PL	138–160	10	6.76
		PLW	138–158	9	5.03
		PUL	138–158	9	6.25
		DU	142–158	6	2.49
SW911	7	PL	156–168	5	2.08
		PLW	156–168	6	2.55
		PUL	156–168	4	2.02
		DU	154–168	4	2.12
SW936	10	PL	94–114	9	3.97
		PLW	92–114	8	4.13
		PUL	94–114	7	3.52
		DU	96–102	4	2.34
SW951	5	PL	123–131	3	1.68
		PLW	123–131	4	2.51
		PUL	123–131	3	1.59
		DU	123–131	4	2.34

**Table 3:** Genetic parameters assessed for 14 STR loci of the study breeds.

Locus	Breeds	H_O_	H_E_	F_IS_	HWE	PIC	PD	P_ID_	PE_1_	PE_2_
S0090	PL	0.659	0.661	0.003	0.0002 ***	0.617	0.839	0.158	0.253	0.427
	PLW	0.737	0.753	0.022	0.0984	0.712	0.899	0.102	0.346	0.524
	PUL	0.626	0.644	0.028	0.0000 ***	0.604	0.823	0.167	0.241	0.417
	DU	0.676	0.619	−0.093	0.0000 ***	0.542	0.712	0.222	0.201	0.340
S0101	PL	0.610	0.634	0.038	0.0000 ***	0.585	0.818	0.183	0.227	0.393
	PLW	0.622	0.690	0.091	0.1991	0.652	0.866	0.138	0.285	0.458
	PUL	0.682	0.706	0.033	0.0001 ***	0.658	0.856	0.135	0.293	0.465
	DU	0.370	0.328	−0.129	0.1360	0.298	0.533	0.482	0.055	0.165
S0155	PL	0.684	0.650	−0.053	0.0196	0.583	0.803	0.189	0.227	0.379
	PLW	0.544	0.610	0.108	0.0098 **	0.534	0.787	0.228	0.187	0.326
	PUL	0.668	0.653	−0.023	1.0	0.591	0.808	0.182	0.231	0.388
	DU	0.630	0.610	−0.032	1.0	0.535	0.763	0.227	0.190	0.330
S0227	PL	0.356	0.341	−0.044	1.0	0.309	0.534	0.466	0.058	0.171
	PLW	0.278	0.296	0.061	1.0	0.275	0.471	0.516	0.044	0.152
	PUL	0.275	0.290	0.080	1.0	0.282	0.464	0.508	0.046	0.159
	DU	0.352	0.350	−0.006	1.0	0.295	0.514	0.478	0.061	0.151
S0228	PL	0.638	0.623	−0.023	1.0	0.586	0.819	0.180	0.225	0.401
	PLW	0.732	0.761	0.038	1.0	0.781	0.936	0.064	0.442	0.620
	PUL	0.607	0.617	0.017	1.0	0.569	0.808	0.194	0.215	0.380
	DU	0.324	0.337	0.038	1.0	0.291	0.507	0.485	0.057	0.153
S0355	PL	0.513	0.552	0.068	0.0044 **	0.495	0.746	0.257	0.164	0.310
	PLW	0.720	0.708	−0.017	0.1680	0.657	0.861	0.136	0.287	0.458
	PUL	0.327	0.452	0.276	0.0000 ***	0.421	0.635	0.332	0.109	0.258
	DU	0.213	0.197	−0.078	0.0970	0.191	0.364	0.651	0.020	0.105
S0386	PL	0.753	0.721	−0.044	0.0032 **	0.688	0.884	0.111	0.326	0.510
	PLW	0.479	0.660	0.274	0.0000 ***	0.603	0.827	0.173	0.242	0.404
	PUL	0.645	0.669	0.037	0.0990	0.638	0.855	0.141	0.273	0.457
	DU	0.474	0.482	0.022	0.3570	0.437	0.675	0.314	0.118	0.260
SW24	PL	0.626	0.650	0.038	0.0046 **	0.598	0.834	0.174	0.241	0.406
	PLW	0.612	0.684	0.105	0.0000 ***	0.635	0.857	0.149	0.275	0.445
	PUL	0.730	0.775	0.058	0.0055 **	0.740	0.912	0.086	0.387	0.564
	DU	0.778	0.746	−0.043	0.0000 ***	0.715	0.892	0.096	0.358	0.541
SW72	PL	0.626	0.650	0.038	0.0074 **	0.598	0.834	0.174	0.241	0.406
	PLW	0.751	0.750	−0.002	1.0	0.710	0.895	0.102	0.348	0.526
	PUL	0.682	0.678	−0.006	1.0	0.621	0.827	0.161	0.261	0.424
	DU	0.769	0.698	−0.117	0.0012 **	0.624	0.809	0.162	0.251	0.410
SW240	bz	0.652	0.645	−0.011	1.00	0.604	0.840	0.167	0.245	0.420
	PLW	0.618	0.634	0.025	1.00	0.610	0.846	0.158	0.249	0.437
	PUL	0.635	0.667	0.048	0.0035 **	0.634	0.846	0.144	0.272	0.455
	DU	0.574	0.599	0.077	0.1820	0.565	0.789	0.200	0.209	0.368
SW857	PL	0.849	0.852	0.004	1.0	0.835	0.958	0.039	0.539	0.703
	PLW	0.830	0.811	−0.027	1.0	0.776	0.934	0.064	0.444	0.621
	PUL	0.844	0.840	−0.005	1.0	0.821	0.950	0.044	0.518	0.686
	DU	0.593	0.599	0.011	0.1890	0.550	0.798	0.210	0.197	0.359
SW911	PL	0.537	0.520	−0.033	1.0	0.434	0.680	0.317	0.137	0.246
	PLW	0.591	0.608	0.027	1.0	0.533	0.773	0.228	0.195	0.334
	PUL	0.564	0.526	−0.105	0.0978	0.436	0.668	0.314	0.131	0.254
	DU	0.593	0.528	−0.122	0.1605	0.483	0.737	0.267	0.146	0.300
SW936	PL	0.738	0.748	0.013	0.0813	0.710	0.892	0.101	0.356	0.533
	PLW	0.811	0.758	−0.070	0.0616	0.720	0.893	0.096	0.361	0.539
	PUL	0.720	0.716	−0.006	0.6825	0.672	0.879	0.125	0.313	0.487
	DU	0.657	0.572	−0.150	0.4658	0.505	0.719	0.250	0.171	0.312
SW951	PL	0.418	0.406	−0.030	1.0	0.366	0.610	0.393	0.082	0.208
	PLW	0.527	0.592	0.121	0.0032 **	0.523	0.782	0.237	0.188	0.322
	PUL	0.398	0.381	−0.062	0.1891	0.337	0.580	0.429	0.069	0.190
	DU	0.647	0.592	−0.093	0.5454	0.505	0.719	0.250	0.171	0.312
$\bar{X}$		0.599	0.605	0.007		0.559	0.771			

**—*p* < 0.01; ***—*p* < 0.001.

**Table 4:** Mean values of genetic parameters assessed for 14 STR loci of the study breeds.

Breeds	H_O_	H_E_	F_IS_	PIC	CPD	CP_ID_	CPE_1_	CPE_2_
PL	0.619	0.618	−0.003	0.581	1 *	1.8 × 10^−11^	0.98413	0.99952
PLW	0.632	0.665	0.054	0.623	1 *	2.0 × 10^−12^	0.99096	0.99978
PUL	0.600	0.616	0.026	0.573	1 *	2.8 × 10^−11^	0.98235	0.99946
DU	0.546	0.520	−0.051	0.477	1 *	6.9 × 10^−9^	0.92755	0.99492

*—actual value < 1.0, equal to approximately > 0.9999999.

## Data Availability

The data presented in this study are available within the article.
